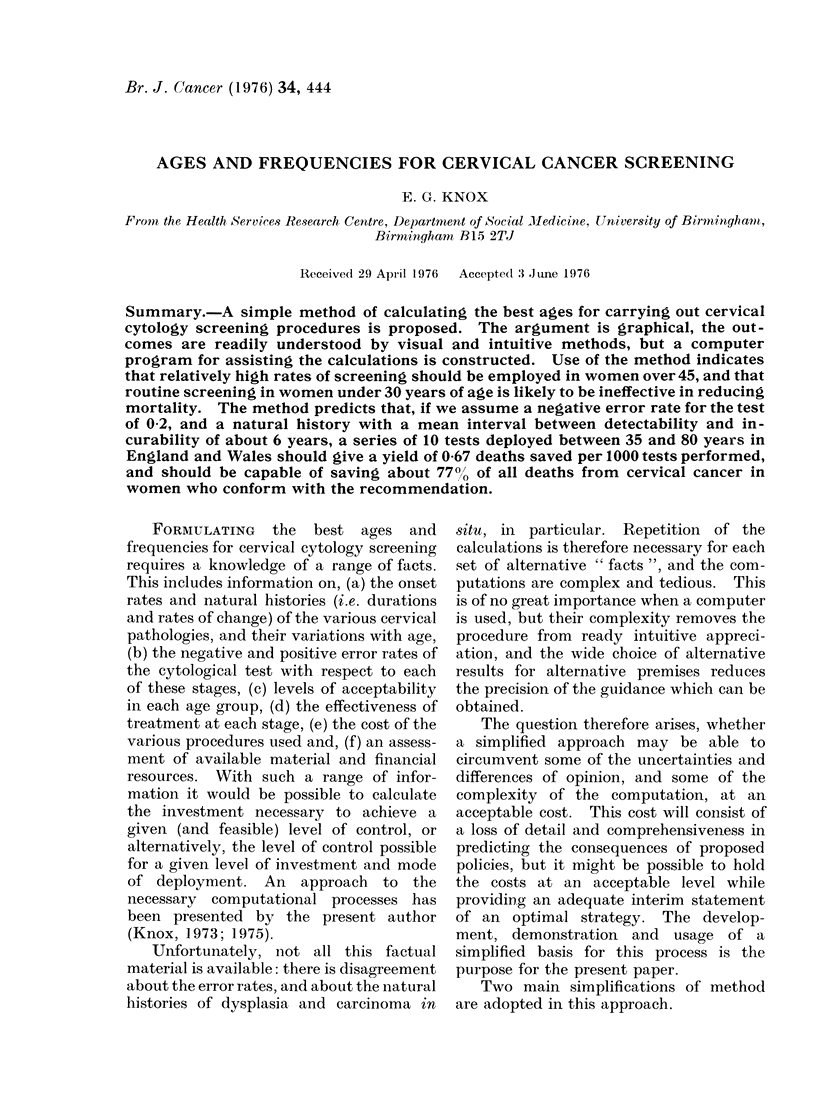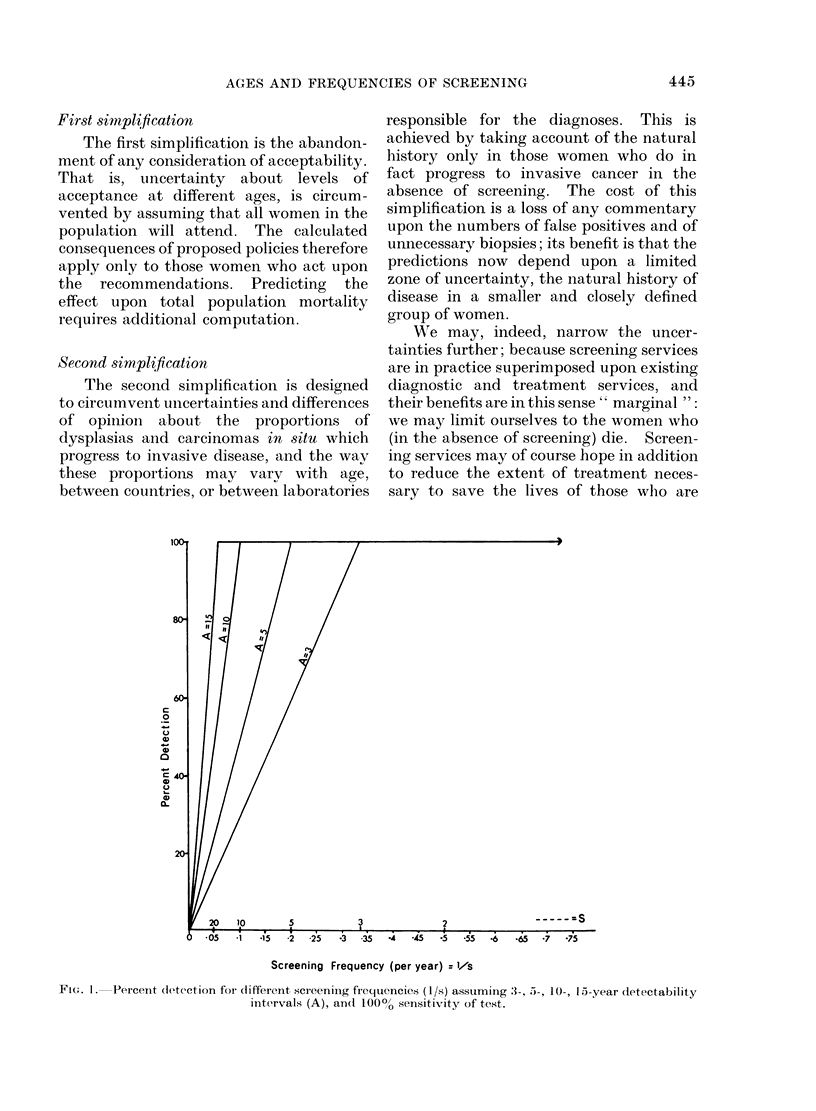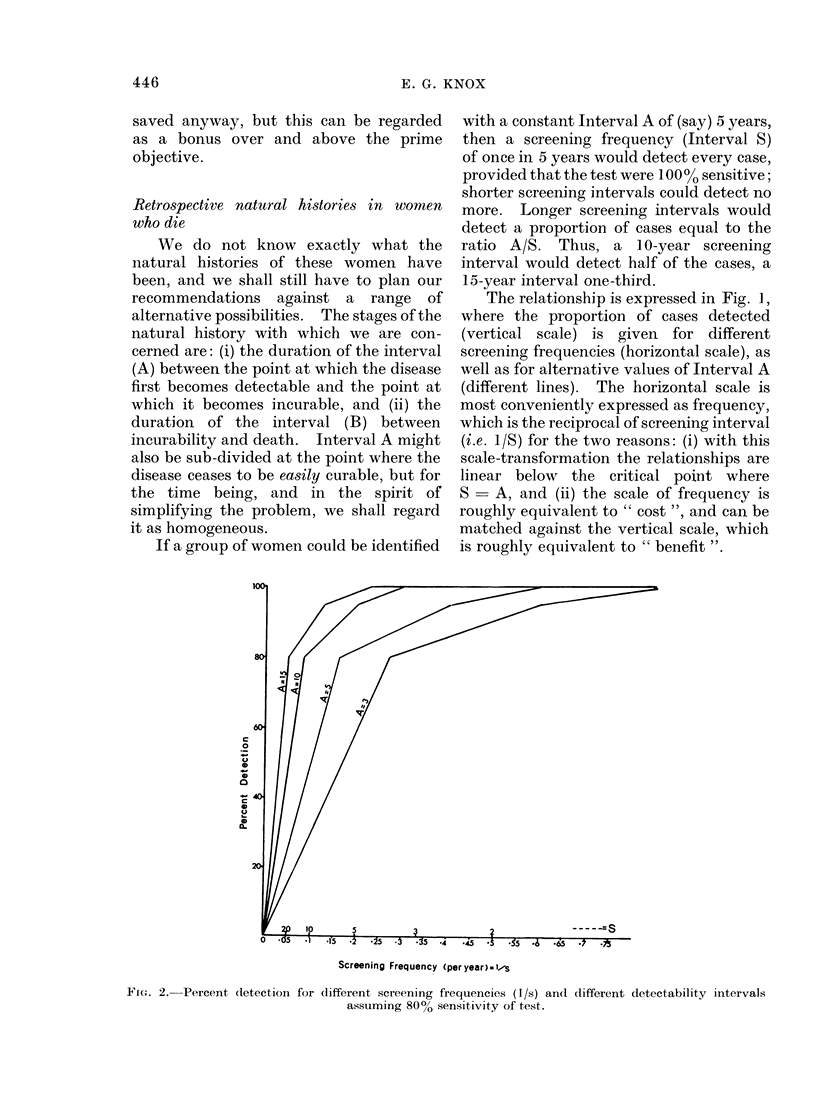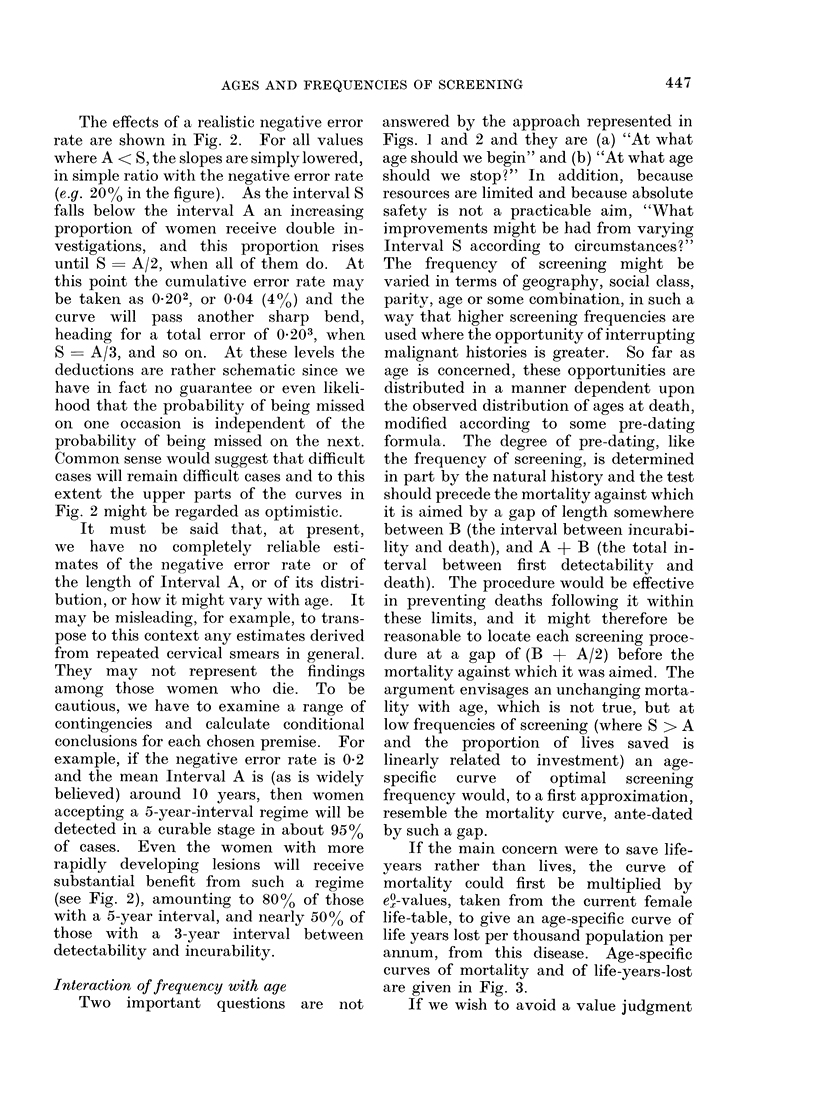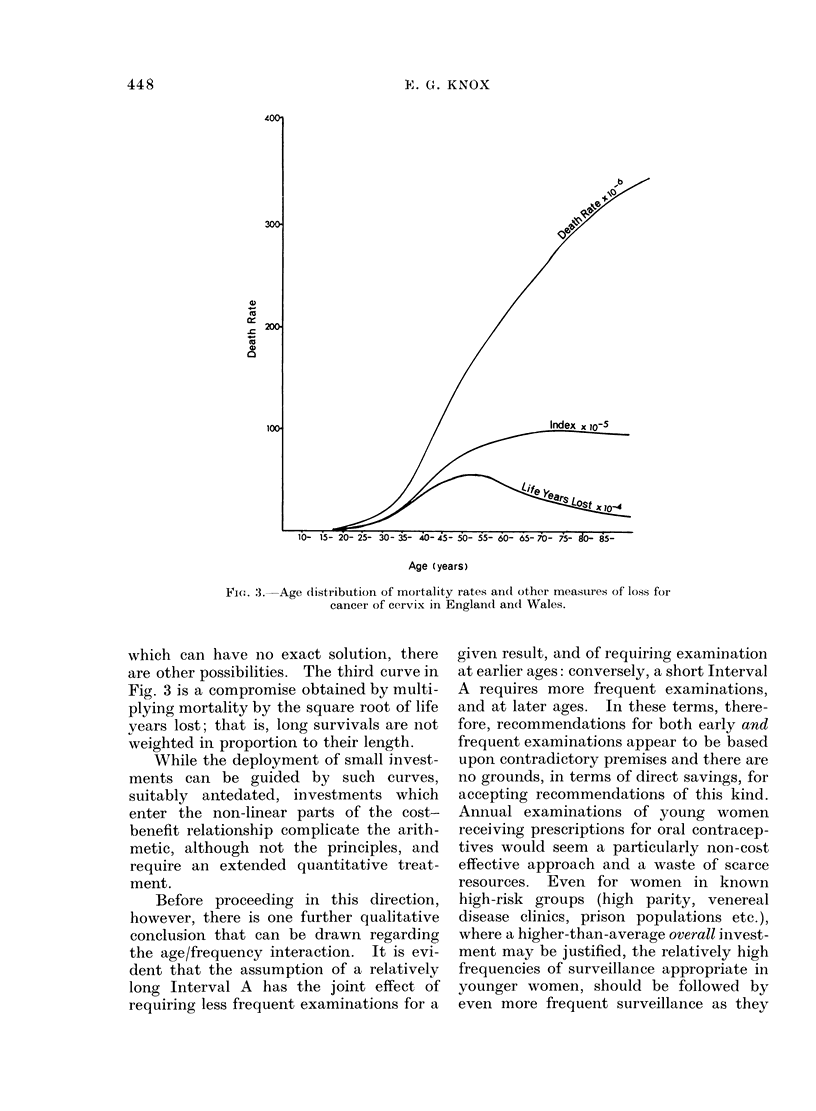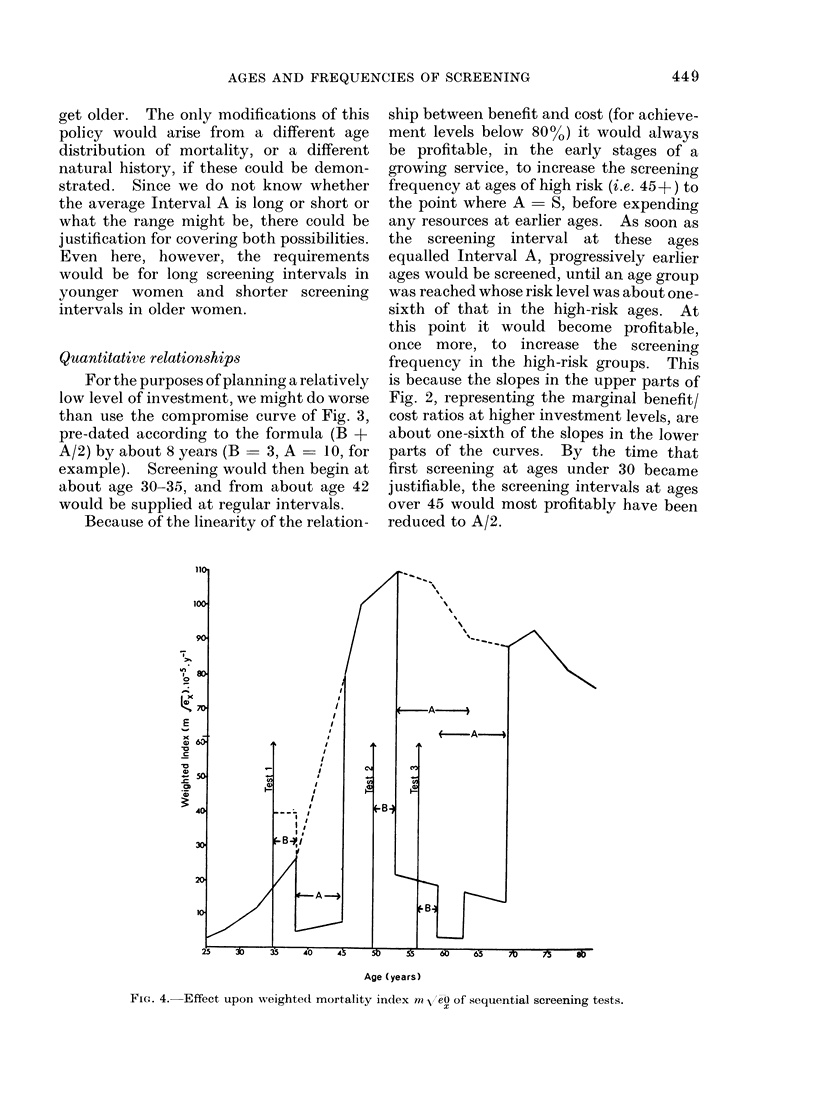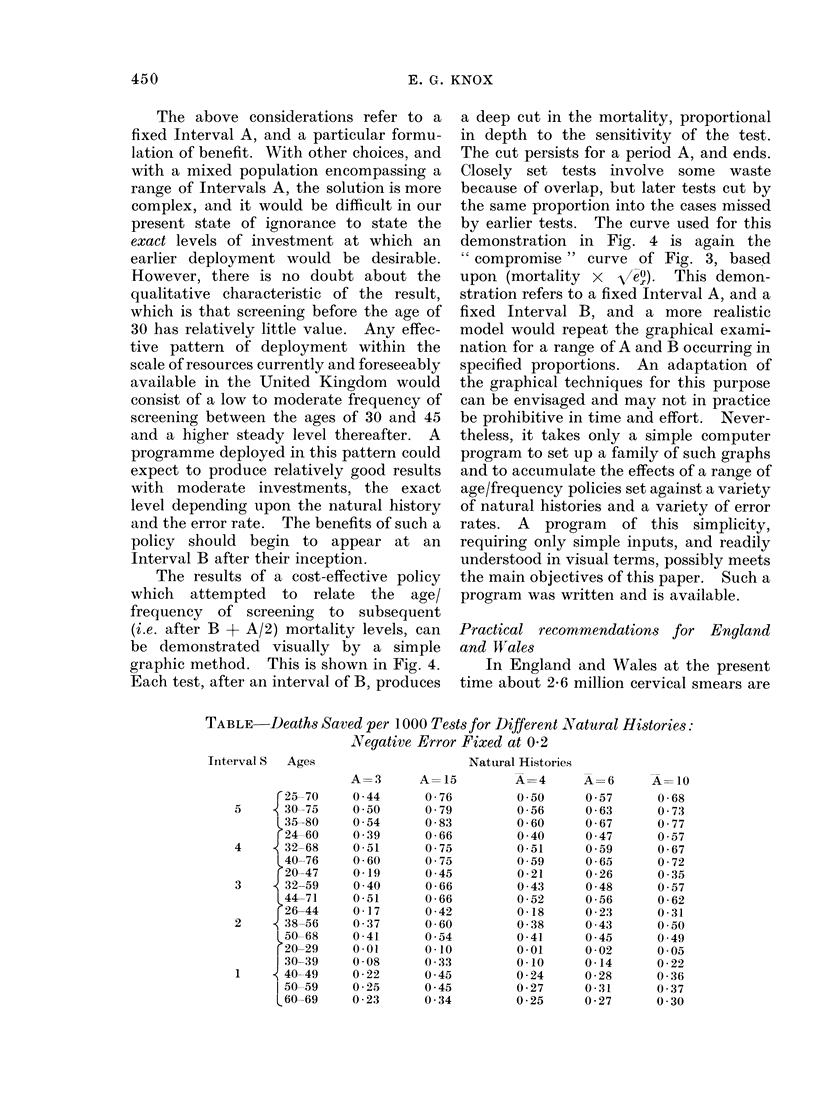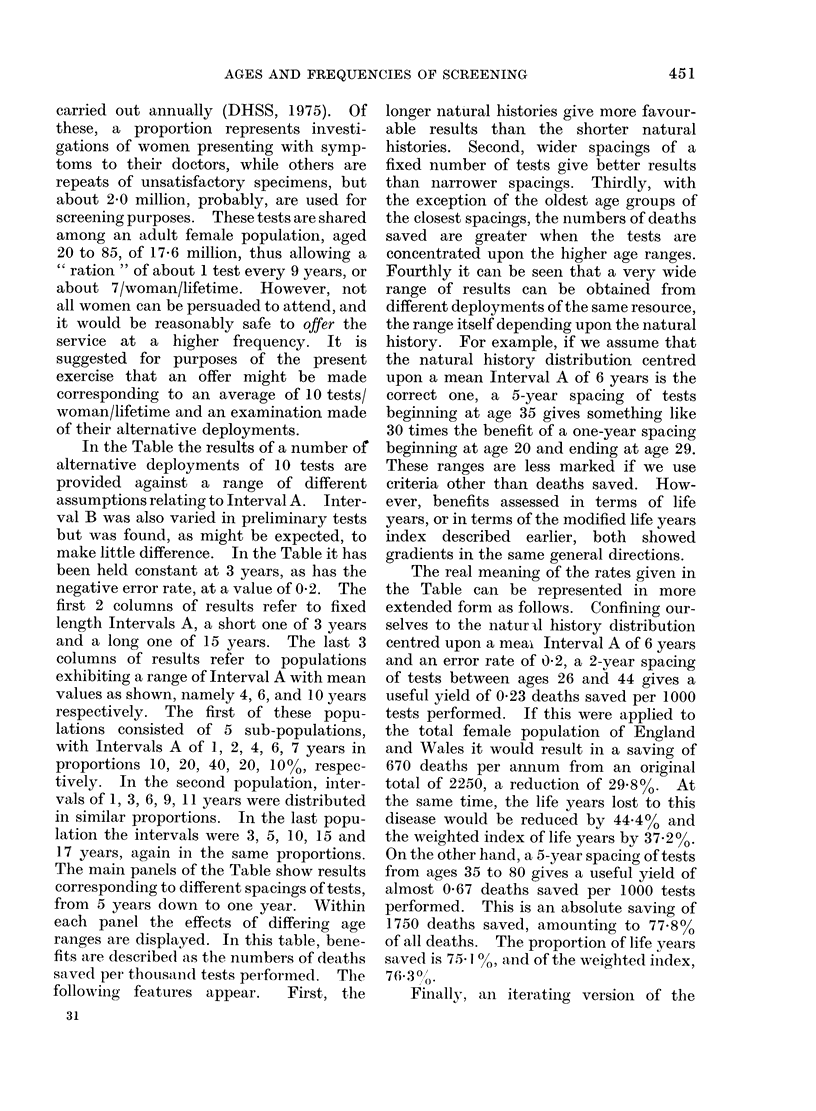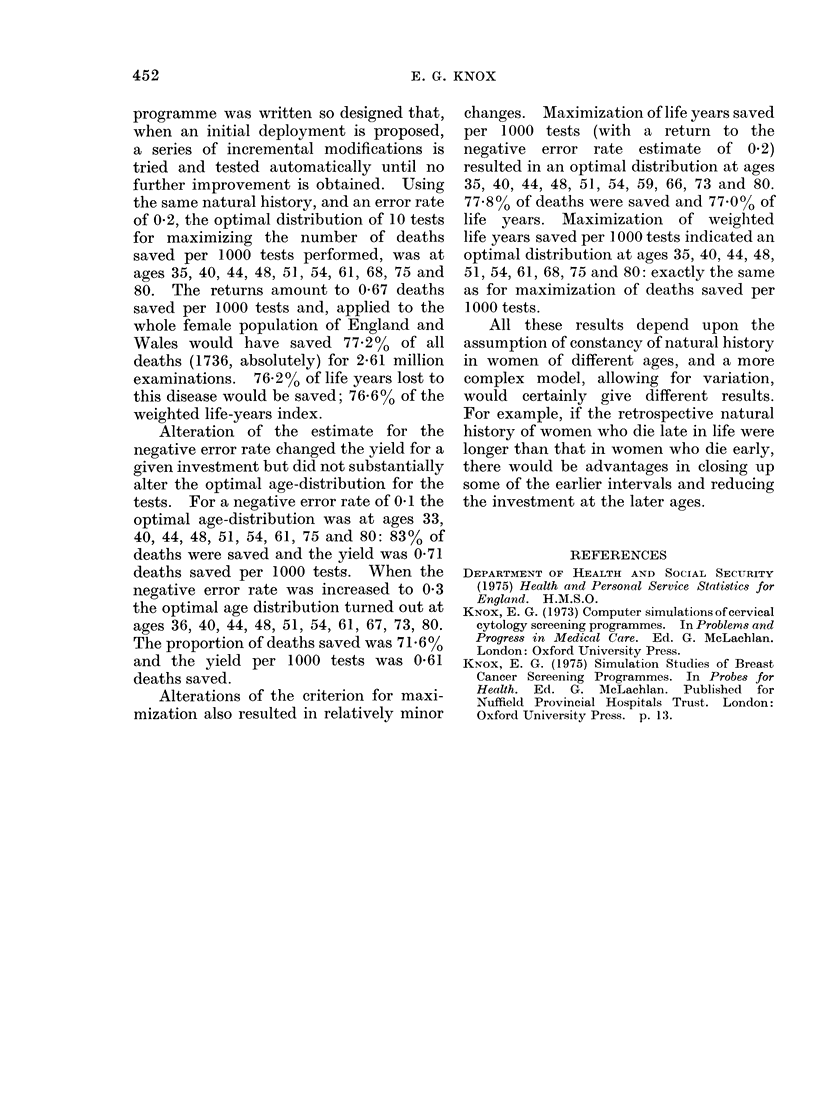# Ages and frequencies for cervical cancer screening.

**DOI:** 10.1038/bjc.1976.190

**Published:** 1976-10

**Authors:** E. G. Knox

## Abstract

A simple method of calculating the best ages for carrying out cervical cytology screening procedures is proposed. The argument is graphical, the outcomes are readily understood by visual and intuitive methods, but a computer program for assisting the calculations is constructed. Use of the method indicates that relatively high rates of screening should be employed in women over 45, and that routine screening in women under 30 years of age is likely to be ineffective in reducing mortality. The method predicts that, if we assume a negative error rate for the test of 0-2, and a natural history with a mean interval between detectability and incurability of about 6 years, a series of 10 tests deployed between 35 and 80 years in England and Wales should give a yield of 0-67 deaths saved per 1000 tests performed, and should be capable of saving about 77% of all deaths from cervical cancer in women who conform with the recommendation.


*Br. J. Cancer* (1976) **34**, 444

### AGES AND FREQUENCIES FOR CERVICAL CANCER SCREENING

E. G. KNOX

*From the Health Services Research Centre, Department of Social Medicine, University of Birmingham, Birmingham B15 2TJ*

Received 29 April 1976 Accepted 3 June 1976

**Summary.—A simple method of calculating the best ages for carrying out cervical cytology screening procedures is proposed. The argument is graphical, the outcomes are readily understood by visual and intuitive methods, but a computer program for assisting the calculations is constructed. Use of the method indicates that relatively high rates of screening should be employed in women over 45, and that routine screening in women under 30 years of age is likely to be ineffective in reducing mortality. The method predicts that, if we assume a negative error rate for the test of 0·2, and a natural history with a mean interval between detectability and incurability of about 6 years, a series of 10 tests deployed between 35 and 80 years in England and Wales should give a yield of 0·67 deaths saved per 1000 tests performed, and should be capable of saving about 77% of all deaths from cervical cancer in women who conform with the recommendation.**

FORMULATING the best ages and frequencies for cervical cytology screening requires a knowledge of a range of facts. This includes information on, (a) the onset rates and natural histories (*i.e.* durations and rates of change) of the various cervical pathologies, and their variations with age, (b) the negative and positive error rates of the cytological test with respect to each of these stages, (c) levels of acceptability in each age group, (d) the effectiveness of treatment at each stage, (e) the cost of the various procedures used and, (f) an assessment of available material and financial resources. With such a range of information it would be possible to calculate the investment necessary to achieve a given (and feasible) level of control, or alternatively, the level of control possible for a given level of investment and mode of deployment. An approach to the necessary computational processes has been presented by the present author (Knox, 1973; 1975).

Unfortunately, not all this factual material is available: there is disagreement about the error rates, and about the natural histories of dysplasia and carcinoma *in situ*, in particular. Repetition of the calculations is therefore necessary for each set of alternative " facts ", and the computations are complex and tedious. This is of no great importance when a computer is used, but their complexity removes the procedure from ready intuitive appreciation, and the wide choice of alternative results for alternative premises reduces the precision of the guidance which can be obtained.

The question therefore arises, whether a simplified approach may be able to circumvent some of the uncertainties and differences of opinion, and some of the complexity of the computation, at an acceptable cost. This cost will consist of a loss of detail and comprehensiveness in predicting the consequences of proposed policies, but it might be possible to hold the costs at an acceptable level while providing an adequate interim statement of an optimal strategy. The development, demonstration and usage of a simplified basis for this process is the purpose for the present paper.

Two main simplifications of method are adopted in this approach.

*First simplification*

The first simplification is the abandonment of any consideration of acceptability. That is, uncertainty about levels of acceptance at different ages, is circumvented by assuming that all women in the population will attend. The calculated consequences of proposed policies therefore apply only to those women who act upon the recommendations. Predicting the effect upon total population mortality requires additional computation.

*Second simplification*

The second simplification is designed to circumvent uncertainties and differences of opinion about the proportions of dysplasias and carcinomas *in situ* which progress to invasive disease, and the way these proportions may vary with age, between countries, or between laboratories responsible for the diagnoses. This is achieved by taking account of the natural history only in those women who do in fact progress to invasive cancer in the absence of screening. The cost of this simplification is a loss of any commentary upon the numbers of false positives and of unnecessary biopsies; its benefit is that the predictions now depend upon a limited zone of uncertainty, the natural history of disease in a smaller and closely defined group of women.

We may, indeed, narrow the uncertainties further; because screening services are in practice superimposed upon existing diagnostic and treatment services, and their benefits are in this sense " marginal ": we may limit ourselves to the women who (in the absence of screening) die. Screening services may of course hope in addition to reduce the extent of treatment necessary to save the lives of those who are

FIG. 1.—Percent detection for different screening frequencies (1/s) assuming 3-, 5-, 10-, 15-year detectability intervals (A), and 100% sensitivity of test.

saved anyway, but this can be regarded as a bonus over and above the prime objective.

*Retrospective natural histories in women who die*

We do not know exactly what the natural histories of these women have been, and we shall still have to plan our recommendations against a range of alternative possibilities. The stages of the natural history with which we are concerned are: (i) the duration of the interval (A) between the point at which the disease first becomes detectable and the point at which it becomes incurable, and (ii) the duration of the interval (B) between incurability and death. Interval A might also be sub-divided at the point where the disease ceases to be *easily* curable, but for the time being, and in the spirit of simplifying the problem, we shall regard it as homogeneous.

If a group of women could be identified with a constant Interval A of (say) 5 years, then a screening frequency (Interval S) of once in 5 years would detect every case, provided that the test were 100% sensitive; shorter screening intervals could detect no more. Longer screening intervals would detect a proportion of cases equal to the ratio A/S. Thus, a 10-year screening interval would detect half of the cases, a 15-year interval one-third.

The relationship is expressed in Fig. 1, where the proportion of cases detected (vertical scale) is given for different screening frequencies (horizontal scale), as well as for alternative values of Interval A (different lines). The horizontal scale is most conveniently expressed as frequency, which is the reciprocal of screening interval (*i.e.* 1/S) for the two reasons: (i) with this scale-transformation the relationships are linear below the critical point where S = A, and (ii) the scale of frequency is roughly equivalent to " cost ", and can be matched against the vertical scale, which is roughly equivalent to " benefit ".

FIG. 2.—Percent detection for different screening frequencies (1/s) and different detectability intervals assuming 80% sensitivity of test.

The effects of a realistic negative error rate are shown in Fig. 2. For all values where $A < S$, the slopes are simply lowered, in simple ratio with the negative error rate (*e.g.* 20% in the figure). As the interval S falls below the interval A an increasing proportion of women receive double investigations, and this proportion rises until $S = A/2$, when all of them do. At this point the cumulative error rate may be taken as $0{\cdot}20^2$, or 0·04 (4%) and the curve will pass another sharp bend, heading for a total error of $0{\cdot}20^3$, when $S = A/3$, and so on. At these levels the deductions are rather schematic since we have in fact no guarantee or even likelihood that the probability of being missed on one occasion is independent of the probability of being missed on the next. Common sense would suggest that difficult cases will remain difficult cases and to this extent the upper parts of the curves in Fig. 2 might be regarded as optimistic.

It must be said that, at present, we have no completely reliable estimates of the negative error rate or of the length of Interval A, or of its distribution, or how it might vary with age. It may be misleading, for example, to transpose to this context any estimates derived from repeated cervical smears in general. They may not represent the findings among those women who die. To be cautious, we have to examine a range of contingencies and calculate conditional conclusions for each chosen premise. For example, if the negative error rate is 0·2 and the mean Interval A is (as is widely believed) around 10 years, then women accepting a 5-year-interval regime will be detected in a curable stage in about 95% of cases. Even the women with more rapidly developing lesions will receive substantial benefit from such a regime (see Fig. 2), amounting to 80% of those with a 5-year interval, and nearly 50% of those with a 3-year interval between detectability and incurability.

*Interaction of frequency with age*

Two important questions are not answered by the approach represented in Figs. 1 and 2 and they are (a) "At what age should we begin" and (b) "At what age should we stop?" In addition, because resources are limited and because absolute safety is not a practicable aim, "What improvements might be had from varying Interval S according to circumstances?" The frequency of screening might be varied in terms of geography, social class, parity, age or some combination, in such a way that higher screening frequencies are used where the opportunity of interrupting malignant histories is greater. So far as age is concerned, these opportunities are distributed in a manner dependent upon the observed distribution of ages at death, modified according to some pre-dating formula. The degree of pre-dating, like the frequency of screening, is determined in part by the natural history and the test should precede the mortality against which it is aimed by a gap of length somewhere between B (the interval between incurability and death), and $A + B$ (the total interval between first detectability and death). The procedure would be effective in preventing deaths following it within these limits, and it might therefore be reasonable to locate each screening procedure at a gap of $(B + A/2)$ before the mortality against which it was aimed. The argument envisages an unchanging mortality with age, which is not true, but at low frequencies of screening (where $S > A$ and the proportion of lives saved is linearly related to investment) an age-specific curve of optimal screening frequency would, to a first approximation, resemble the mortality curve, ante-dated by such a gap.

If the main concern were to save life-years rather than lives, the curve of mortality could first be multiplied by $e_x^0$-values, taken from the current female life-table, to give an age-specific curve of life years lost per thousand population per annum, from this disease. Age-specific curves of mortality and of life-years-lost are given in Fig. 3.

If we wish to avoid a value judgment

FIG. 3.—Age distribution of mortality rates and other measures of loss for cancer of cervix in England and Wales.

which can have no exact solution, there are other possibilities. The third curve in Fig. 3 is a compromise obtained by multiplying mortality by the square root of life years lost; that is, long survivals are not weighted in proportion to their length.

While the deployment of small investments can be guided by such curves, suitably antedated, investments which enter the non-linear parts of the cost–benefit relationship complicate the arithmetic, although not the principles, and require an extended quantitative treatment.

Before proceeding in this direction, however, there is one further qualitative conclusion that can be drawn regarding the age/frequency interaction. It is evident that the assumption of a relatively long Interval A has the joint effect of requiring less frequent examinations for a given result, and of requiring examination at earlier ages: conversely, a short Interval A requires more frequent examinations, and at later ages. In these terms, therefore, recommendations for both early *and* frequent examinations appear to be based upon contradictory premises and there are no grounds, in terms of direct savings, for accepting recommendations of this kind. Annual examinations of young women receiving prescriptions for oral contraceptives would seem a particularly non-cost effective approach and a waste of scarce resources. Even for women in known high-risk groups (high parity, venereal disease clinics, prison populations etc.), where a higher-than-average *overall* investment may be justified, the relatively high frequencies of surveillance appropriate in younger women, should be followed by even more frequent surveillance as they get older. The only modifications of this policy would arise from a different age distribution of mortality, or a different natural history, if these could be demonstrated. Since we do not know whether the average Interval A is long or short or what the range might be, there could be justification for covering both possibilities. Even here, however, the requirements would be for long screening intervals in younger women and shorter screening intervals in older women.

*Quantitative relationships*

For the purposes of planning a relatively low level of investment, we might do worse than use the compromise curve of Fig. 3, pre-dated according to the formula (B + A/2) by about 8 years (B = 3, A = 10, for example). Screening would then begin at about age 30–35, and from about age 42 would be supplied at regular intervals.

Because of the linearity of the relationship between benefit and cost (for achievement levels below 80%) it would always be profitable, in the early stages of a growing service, to increase the screening frequency at ages of high risk (*i.e.* 45+) to the point where A = S, before expending any resources at earlier ages. As soon as the screening interval at these ages equalled Interval A, progressively earlier ages would be screened, until an age group was reached whose risk level was about one-sixth of that in the high-risk ages. At this point it would become profitable, once more, to increase the screening frequency in the high-risk groups. This is because the slopes in the upper parts of Fig. 2, representing the marginal benefit/cost ratios at higher investment levels, are about one-sixth of the slopes in the lower parts of the curves. By the time that first screening at ages under 30 became justifiable, the screening intervals at ages over 45 would most profitably have been reduced to A/2.

FIG. 4.—Effect upon weighted mortality index $m\sqrt{e_x^0}$ of sequential screening tests.

The above considerations refer to a fixed Interval A, and a particular formulation of benefit. With other choices, and with a mixed population encompassing a range of Intervals A, the solution is more complex, and it would be difficult in our present state of ignorance to state the *exact* levels of investment at which an earlier deployment would be desirable. However, there is no doubt about the qualitative characteristic of the result, which is that screening before the age of 30 has relatively little value. Any effective pattern of deployment within the scale of resources currently and foreseeably available in the United Kingdom would consist of a low to moderate frequency of screening between the ages of 30 and 45 and a higher steady level thereafter. A programme deployed in this pattern could expect to produce relatively good results with moderate investments, the exact level depending upon the natural history and the error rate. The benefits of such a policy should begin to appear at an Interval B after their inception.

The results of a cost-effective policy which attempted to relate the age/frequency of screening to subsequent (*i.e.* after B + A/2) mortality levels, can be demonstrated visually by a simple graphic method. This is shown in Fig. 4. Each test, after an interval of B, produces a deep cut in the mortality, proportional in depth to the sensitivity of the test. The cut persists for a period A, and ends. Closely set tests involve some waste because of overlap, but later tests cut by the same proportion into the cases missed by earlier tests. The curve used for this demonstration in Fig. 4 is again the "compromise" curve of Fig. 3, based upon (mortality $\times$ $\sqrt{\mathring{e}_x}$). This demonstration refers to a fixed Interval A, and a fixed Interval B, and a more realistic model would repeat the graphical examination for a range of A and B occurring in specified proportions. An adaptation of the graphical techniques for this purpose can be envisaged and may not in practice be prohibitive in time and effort. Nevertheless, it takes only a simple computer program to set up a family of such graphs and to accumulate the effects of a range of age/frequency policies set against a variety of natural histories and a variety of error rates. A program of this simplicity, requiring only simple inputs, and readily understood in visual terms, possibly meets the main objectives of this paper. Such a program was written and is available.

*Practical recommendations for England and Wales*

In England and Wales at the present time about 2·6 million cervical smears are

TABLE—*Deaths Saved per* 1000 *Tests for Different Natural Histories: Negative Error Fixed at* 0·2

| Interval S | Ages | A = 3 | A = 15 | $\bar{A}$ = 4 | $\bar{A}$ = 6 | $\bar{A}$ = 10 |
|---|---|---|---|---|---|---|
| | | | Natural Histories | | | |
| 5 | 25–70 | 0·44 | 0·76 | 0·50 | 0·57 | 0·68 |
| | 30–75 | 0·50 | 0·79 | 0·56 | 0·63 | 0·73 |
| | 35–80 | 0·54 | 0·83 | 0·60 | 0·67 | 0·77 |
| 4 | 24–60 | 0·39 | 0·66 | 0·40 | 0·47 | 0·57 |
| | 32–68 | 0·51 | 0·75 | 0·51 | 0·59 | 0·67 |
| | 40–76 | 0·60 | 0·75 | 0·59 | 0·65 | 0·72 |
| 3 | 20–47 | 0·19 | 0·45 | 0·21 | 0·26 | 0·35 |
| | 32–59 | 0·40 | 0·66 | 0·43 | 0·48 | 0·57 |
| | 44–71 | 0·51 | 0·66 | 0·52 | 0·56 | 0·62 |
| 2 | 26–44 | 0·17 | 0·42 | 0·18 | 0·23 | 0·31 |
| | 38–56 | 0·37 | 0·60 | 0·38 | 0·43 | 0·50 |
| | 50–68 | 0·41 | 0·54 | 0·41 | 0·45 | 0·49 |
| 1 | 20–29 | 0·01 | 0·10 | 0·01 | 0·02 | 0·05 |
| | 30–39 | 0·08 | 0·33 | 0·10 | 0·14 | 0·22 |
| | 40–49 | 0·22 | 0·45 | 0·24 | 0·28 | 0·36 |
| | 50–59 | 0·25 | 0·45 | 0·27 | 0·31 | 0·37 |
| | 60–69 | 0·23 | 0·34 | 0·25 | 0·27 | 0·30 |

carried out annually (DHSS, 1975). Of these, a proportion represents investigations of women presenting with symptoms to their doctors, while others are repeats of unsatisfactory specimens, but about 2·0 million, probably, are used for screening purposes. These tests are shared among an adult female population, aged 20 to 85, of 17·6 million, thus allowing a " ration " of about 1 test every 9 years, or about 7/woman/lifetime. However, not all women can be persuaded to attend, and it would be reasonably safe to *offer* the service at a higher frequency. It is suggested for purposes of the present exercise that an offer might be made corresponding to an average of 10 tests/woman/lifetime and an examination made of their alternative deployments.

In the Table the results of a number of alternative deployments of 10 tests are provided against a range of different assumptions relating to Interval A. Interval B was also varied in preliminary tests but was found, as might be expected, to make little difference. In the Table it has been held constant at 3 years, as has the negative error rate, at a value of 0·2. The first 2 columns of results refer to fixed length Intervals A, a short one of 3 years and a long one of 15 years. The last 3 columns of results refer to populations exhibiting a range of Interval A with mean values as shown, namely 4, 6, and 10 years respectively. The first of these populations consisted of 5 sub-populations, with Intervals A of 1, 2, 4, 6, 7 years in proportions 10, 20, 40, 20, 10%, respectively. In the second population, intervals of 1, 3, 6, 9, 11 years were distributed in similar proportions. In the last population the intervals were 3, 5, 10, 15 and 17 years, again in the same proportions. The main panels of the Table show results corresponding to different spacings of tests, from 5 years down to one year. Within each panel the effects of differing age ranges are displayed. In this table, benefits are described as the numbers of deaths saved per thousand tests performed. The following features appear. First, the longer natural histories give more favourable results than the shorter natural histories. Second, wider spacings of a fixed number of tests give better results than narrower spacings. Thirdly, with the exception of the oldest age groups of the closest spacings, the numbers of deaths saved are greater when the tests are concentrated upon the higher age ranges. Fourthly it can be seen that a very wide range of results can be obtained from different deployments of the same resource, the range itself depending upon the natural history. For example, if we assume that the natural history distribution centred upon a mean Interval A of 6 years is the correct one, a 5-year spacing of tests beginning at age 35 gives something like 30 times the benefit of a one-year spacing beginning at age 20 and ending at age 29. These ranges are less marked if we use criteria other than deaths saved. However, benefits assessed in terms of life years, or in terms of the modified life years index described earlier, both showed gradients in the same general directions.

The real meaning of the rates given in the Table can be represented in more extended form as follows. Confining ourselves to the natural history distribution centred upon a mean Interval A of 6 years and an error rate of 0·2, a 2-year spacing of tests between ages 26 and 44 gives a useful yield of 0·23 deaths saved per 1000 tests performed. If this were applied to the total female population of England and Wales it would result in a saving of 670 deaths per annum from an original total of 2250, a reduction of 29·8%. At the same time, the life years lost to this disease would be reduced by 44·4% and the weighted index of life years by 37·2%. On the other hand, a 5-year spacing of tests from ages 35 to 80 gives a useful yield of almost 0·67 deaths saved per 1000 tests performed. This is an absolute saving of 1750 deaths saved, amounting to 77·8% of all deaths. The proportion of life years saved is 75·1%, and of the weighted index, 76·3%.

Finally, an iterating version of the programme was written so designed that, when an initial deployment is proposed, a series of incremental modifications is tried and tested automatically until no further improvement is obtained. Using the same natural history, and an error rate of 0·2, the optimal distribution of 10 tests for maximizing the number of deaths saved per 1000 tests performed, was at ages 35, 40, 44, 48, 51, 54, 61, 68, 75 and 80. The returns amount to 0·67 deaths saved per 1000 tests and, applied to the whole female population of England and Wales would have saved 77·2% of all deaths (1736, absolutely) for 2·61 million examinations. 76·2% of life years lost to this disease would be saved; 76·6% of the weighted life-years index.

Alteration of the estimate for the negative error rate changed the yield for a given investment but did not substantially alter the optimal age-distribution for the tests. For a negative error rate of 0·1 the optimal age-distribution was at ages 33, 40, 44, 48, 51, 54, 61, 75 and 80: 83% of deaths were saved and the yield was 0·71 deaths saved per 1000 tests. When the negative error rate was increased to 0·3 the optimal age distribution turned out at ages 36, 40, 44, 48, 51, 54, 61, 67, 73, 80. The proportion of deaths saved was 71·6% and the yield per 1000 tests was 0·61 deaths saved.

Alterations of the criterion for maximization also resulted in relatively minor changes. Maximization of life years saved per 1000 tests (with a return to the negative error rate estimate of 0·2) resulted in an optimal distribution at ages 35, 40, 44, 48, 51, 54, 59, 66, 73 and 80. 77·8% of deaths were saved and 77·0% of life years. Maximization of weighted life years saved per 1000 tests indicated an optimal distribution at ages 35, 40, 44, 48, 51, 54, 61, 68, 75 and 80: exactly the same as for maximization of deaths saved per 1000 tests.

All these results depend upon the assumption of constancy of natural history in women of different ages, and a more complex model, allowing for variation, would certainly give different results. For example, if the retrospective natural history of women who die late in life were longer than that in women who die early, there would be advantages in closing up some of the earlier intervals and reducing the investment at the later ages.

### REFERENCES

Department of Health and Social Security (1975) *Health and Personal Service Statistics for England.* H.M.S.O.

Knox, E. G. (1973) Computer simulations of cervical cytology screening programmes. In *Problems and Progress in Medical Care.* Ed. G. McLachlan. London: Oxford University Press.

Knox, E. G. (1975) Simulation Studies of Breast Cancer Screening Programmes. In *Probes for Health.* Ed. G. McLachlan. Published for Nuffield Provincial Hospitals Trust. London: Oxford University Press. p. 13.